# Peripheral blood lymphocyte subsets predict the efficacy of TACE with or without PD-1 inhibitors in patients with hepatocellular carcinoma: a prospective clinical study

**DOI:** 10.3389/fimmu.2024.1325330

**Published:** 2024-02-09

**Authors:** Hongyu Wang, Huijie Huang, Ting Liu, Yaoming Chen, Jinwei Li, Min He, Jianxin Peng, Enyu Liang, Jiaping Li, Wendao Liu

**Affiliations:** ^1^Department of Interventional Therapy, The Second Affiliated Hospital of Guangzhou University of Chinese Medicine, Guangzhou, China; ^2^Guangdong Provincial Key laboratory of Chinese Medicine for Prevention and Treatment of Refractory Chronic Diseases, The Second Affiliated Hospital of Guangzhou University of Chinese Medicine, Guangzhou, China; ^3^Department of Laboratory Medicine, The Second Clinical College of Guangzhou University of Chinese Medicine, Guangzhou, China; ^4^State Key Laboratory of Traditional Chinese Medicine Syndrome, The Second Affiliated Hospital of Guangzhou University of Chinese Medicine, Guangzhou, China; ^5^Department of Laboratory Medicine, The First Affiliated Hospital of Sun Yat-sen University, Guangzhou, China; ^6^Department of Hepatobiliary Surgery, The Second Affiliated Hospital of Guangzhou University of Chinese Medicine, Guangzhou, China; ^7^Department of Interventional Oncology, The First Affiliated Hospital of Sun Yat-Sen University, Guangzhou, China

**Keywords:** lymphocyte subsets, PD-1+ T cells, prognosis, hepatocellular carcinoma, PD-1 inhibitors

## Abstract

**Background:**

Although peripheral blood lymphocyte subsets, particularly PD-1+ T cells, are promising prognostic indicators for patients with cancer. However, their clinical significance remains unclear.

**Methods:**

We prospectively enrolled 157 patients with hepatocellular carcinoma (HCC) treated with transcatheter arterial chemoembolization combined with or without PD-1 inhibitors. Twenty peripheral lymphocyte subsets and cytokines were analyzed. We analyzed the differences in PD-1+ T cells between patients treated with and without PD-1 inhibitors and their associations with tumor response, survival prognosis, and clinical features.

**Results:**

We found that the baseline CD8+PD-1+ and CD4+PD-1+ T-cell frequencies in patients who had received PD-1 inhibitors were lower than those in patients who had not received PD-1 inhibitors (p < 0.001). In the former patients, there were no differences in PD-1+ T-cell frequencies between the responder and non-responder subgroups (p > 0.05), whereas in the latter patients, the levels of CD8+PD-1+ T cells, CD4+PD-1+ T cells, and CD8+PD-1+/CD4+PD-1+ ratio did not predict tumor response, progression-free survival (PFS), or overall survival (OS) (p>0.05). Furthermore, in multivariate analysis of patients treated with or without PD-1 inhibitors revealed that the levels of CD8+CD38+ T cells (OR = 2.806, p = 0.006) were associated with tumor response, whereas those of CD8+CD28+ T cells (p = 0.038, p = 0.001) and natural killer (NK) cells (p = 0.001, p = 0.027) were associated with PFS and OS. Although, these independent prognostic factors were associated with progressive tumor characteristics (p<0.05), with the exception of CD8+CD28+ T cells, changes in these factors before and after treatment were unassociated with tumor response (p > 0.05).

**Conclusion:**

Circulating CD8+CD38+ T cells, CD8+CD28+ T cells, and NK cells were identified as potential prognostic factors for tumor response and survival in patients with HCC. Contrastingly, although PD-1 inhibitors can effectively block the T cell PD-1 receptor, the baseline PD-1+ T-cell frequencies and changes in the frequency of these cells have limited prognostic value.

## 1 Introduction

Hepatocellular carcinoma (HCC), which accounts for 75%–85% of primary liver cancers, is among the most prevalent and fatal malignancies worldwide (1). Transcatheter arterial chemoembolization (TACE), the first-line treatment for patients with unresectable intermediate-stage HCC (2), is effective in patients with early- or advanced-stage HCC (3), and compared with monotherapy based on TACE or tyrosine kinase inhibitors (TKIs), TACE plus TKIs has been established to improve clinical outcomes for unresectable HCC (4, 5). Recent research has shown that the clinical benefit of triple combination therapy comprising TACE+TKIs+programmed cell death (PD)-1/programmed death-ligand 1 (PD-L1) inhibitors are significantly superior to those of dual combination therapy comprising TACE and TKIs (6–8). TKIs regulate the tumor immune microenvironment (9, 10). PD-1 inhibitors block the PD-1 receptors on the surface of T cells, prevent the binding of PD-1 to PD-L1 on the tumor surface, and activate the anti-tumor immunity of cytotoxic T cells (11). Although in recent years, predictive biomarkers based on PD-1/PD-L1 expression, tumor-infiltrating lymphocytes (TILs), and the genetic characteristics of tumor tissue have been reported (12, 13), these markers have yet to be widely validated or used to predict clinical benefits, and thus clinical risk factors still serve as a foundation for treatment choices.

Whereas most of the relevant studies conducted to date have tended to focus on PD-L1 expression in tumor cells and macrophages in the tumor microenvironment, the expression of PD-1 in peripheral T cells has been studied to a notably lesser extent. PD-1, an immune checkpoint receptor, is highly expressed on the surface of functionally exhausted T cells in response to persistent antigen stimulation in patients with tumors or chronic infections. This may explain the association between high levels of PD-1 expression on peripheral blood CD3+ T cells and CD8+ T cells and poor overall survival (OS) and progression-free survival (PFS) in patients with advanced-stage non-small cell lung cancer (NSCLC) treated with nivolumab (14). However, the findings of a further study have indicated that high levels of circulating CD8+PD-1+ T cells have a positive influence on the prognosis of patients with immune checkpoint inhibitor (ICI)-treated advanced NSCLC (15). Moreover, it has been demonstrated that HCC patients with high levels of circulating CD4+PD-1+ T cells are more likely to respond to tremelimumab therapy (16). Consequently, it has yet to be sufficiently established whether the circulating PD-1+ T-cell frequency and its change in response to ICI therapy have any prognostic value.

TILs influence the behavior of human tumors, and the relative abundance and phenotypes of specific subsets of TILs have been extensively investigated as potential biomarkers for ICI treatment (17–19). However, for many patients with advanced liver cancer, the detection of TILs is not feasible, owing to the limited availability of tumor tissues. In this regard, peripheral blood lymphocyte subsets have been identified as promising biomarkers for characterizing differences between cancer patients and healthy individuals, predicting patient prognosis, and determining treatment strategies. Nevertheless, the results of these studies have tended to be inconsistent. In theory, natural killer (NK) cells and CD8+ T cells are cytotoxic; however, the frequencies of circulating NK cells and CD8+ T cells are lower and higher, respectively, in patients with liver cancer than in healthy individuals (20, 21). Conversely, the findings of other studies have revealed reductions in the proportions of NK cells and CD8+ T cells in patients with cancer, whereas there is an increase in the CD4+/CD8+ T-cell ratio (22, 23). Although it is generally believed that high levels of NK, CD4+ T, and CD8+ T cells predict better tumor response and prolonged PFS in patients with NSCLC (23, 24), the findings of one study have indicated that high baseline NK cell levels in patients with advanced NSCLC treated with nivolumab are associated with a poor prognosis (14). These authors also reported that high levels of CD8+ T cells are associated with prolonged OS and PFS, whereas the frequency of CD8+ T cells in patients with tumor progression was higher than that in patients in a clinical benefit group (14). Moreover, different studies have reported variable predictive efficacies for T-cell functional subsets characterized by CD28 and CD38 expression (25–27).

In our previous study, we established that conventional circulating lymphocyte subsets were generally ineffective as prognostic predictors for HCC patients treated with TACE (28). In this prospective cohort study, we accordingly sought to characterize baseline circulating lymphocyte subsets and their changes in HCC patients treated with TACE administered with or without PD-1 inhibitors using high-dimensional flow cytometry and attempted to identify effective prognostic biomarkers.

## 2 Materials and methods

### 2.1 Patients

This study was approved by the Ethics Committee of the First Affiliated Hospital of Sun Yat-Sen University. All patients were informed of the study’s aims and procedures and consented to enrollment. The cohort included as many patients as possible with a clinical or pathological diagnosis of HCC, covering patients with BCLC stages A, B, and C, as well as those who had received previous treatment. Patients with an Eastern Cooperative Oncology Group performance status score greater than 3, a Child-Pugh score greater than 13, or obvious infective symptoms were excluded. At enrollment, peripheral blood was drawn from patients prior to treatment to assess baseline levels of lymphocyte subsets and the cytokines IL-6 and IFN-γ. The outcomes of these preliminary analyses did not influence decisions regarding individual treatment plans.

### 2.2 Treatment and follow-up

On the basis of the characteristics and staging of tumors, we recommend TACE as the basic local treatment, and/or combination systemic therapy, such as TKIs and PD-1 inhibitors. TACE procedures were based on super-selective techniques and an operational protocol described in our previous study (28). The TKIs used at our research center included first-line drugs, such as sorafenib and lenvatinib, and second-line drugs, such as regorafenib and apatinib. PD-1 inhibitors include camrelizumab, sintilimab, tislelizumab. The treatment protocols adopted in this study all comply with Chinese clinical guidelines for the management of HCC (29); however, due to cost and poor compliance, some patients have received relatively conservative treatment or in some cases, the use of TKIs or ICIs bas been delayed.

To evaluate tumor response and determine subsequent treatment plans, patients underwent an initial enhanced CT or MRI examination 4 to 8 weeks after the preliminary treatment. Subsequent follow-up intervals were typically between 1 and 3 months. A tumor response 3 months after the initial treatment was evaluated based on modified RECIST (mRECIST). Responders were defined as those patients with a confirmed complete response (CR) or partial response (PR), whereas non-responders were defined as patients with confirmed stable disease (SD) or progressive disease (PD). PFS was defined from the date of initial lymphocyte subset detection initiation to tumor progression or death due to any cause in the absence of progression. OS was defined from the date of initial lymphocyte subset detection initiation to the data of death due to any cause.

### 2.3 Detection of lymphocyte subsets and cytokines

At enrollment, three samples of venous blood (two EDTA anticoagulant tubes, one separation gel coagulation promoting tube) were collected from patients prior treatment. Fresh blood samples were delivered to our clinical laboratory within 4 hours of collection. CD3+ T, CD4+ T, CD8+ T, CD19+ B, and CD16+ CD56+ NK cells were stained using BD Multitest 6-color TBNK reagent in Trucount tubes (Cat:662997). The inhibitory and activated T lymphocyte subsets were also analyzed based on a single-platform technique by ten-color flow cytometry. The data were collected and analyzed on a BD FACS Canto II flow cytometer. The main antibodies were CD45 KrO (B36294), CD3 PB (B49204), CD4 APC-cy7 (341115), CD8 PE-cy7 (664999), PD-1 Percp-cy5.5 (561273), CD28 PE (662797), CD38 APC (345807), HLA-DR FITC (652827). The gating strategy is shown in **Supplementary Figure 1**. The concentrations of the cytokines IL-6 and IFN-γ were determined using enzyme-linked immunosorbent assays (Hangzhou Clongene Biotech Co. Ltd., China). The procedures were performed in accordance with the manufacturer’s protocols. Additionally, after 4-8 weeks of enrollment, the peripheral blood of patients was collected again to detect the aforementioned immune indicators and evaluate the clinical significance of any changes.

### 2.4 Statistical analysis

Continuous variables and categorical variables are presented as the means and standard deviations, or medians and interquartile ranges, and were compared between groups using Student’s *t*-test, the Mann-Whitney U test, a paired *t*-test, or the chi-square test. The cutoff values for lymphocyte subsets and cytokines for predicting a tumor response were determined by receiver operating characteristic (ROC) curve analysis. Logistic regression was performed to identify variables associated with tumor response. Univariate and multivariate Cox analyses were also conducted to identify variables associated with survival outcomes, and only factors that reached a significance threshold of p < 0.1 in univariate analysis were selected for multivariate analysis. All presented p-values are two-sided, and a p-value < 0.05 was considered to indicate a statistical significance. IBM SPSS version 24.0 was used for statistical analysis, and GraphPad Prism version 8.0.1 was used for graphical presentation of the data.

## 3 Results

### 3.1 Patient characteristics

**Figure 1:**
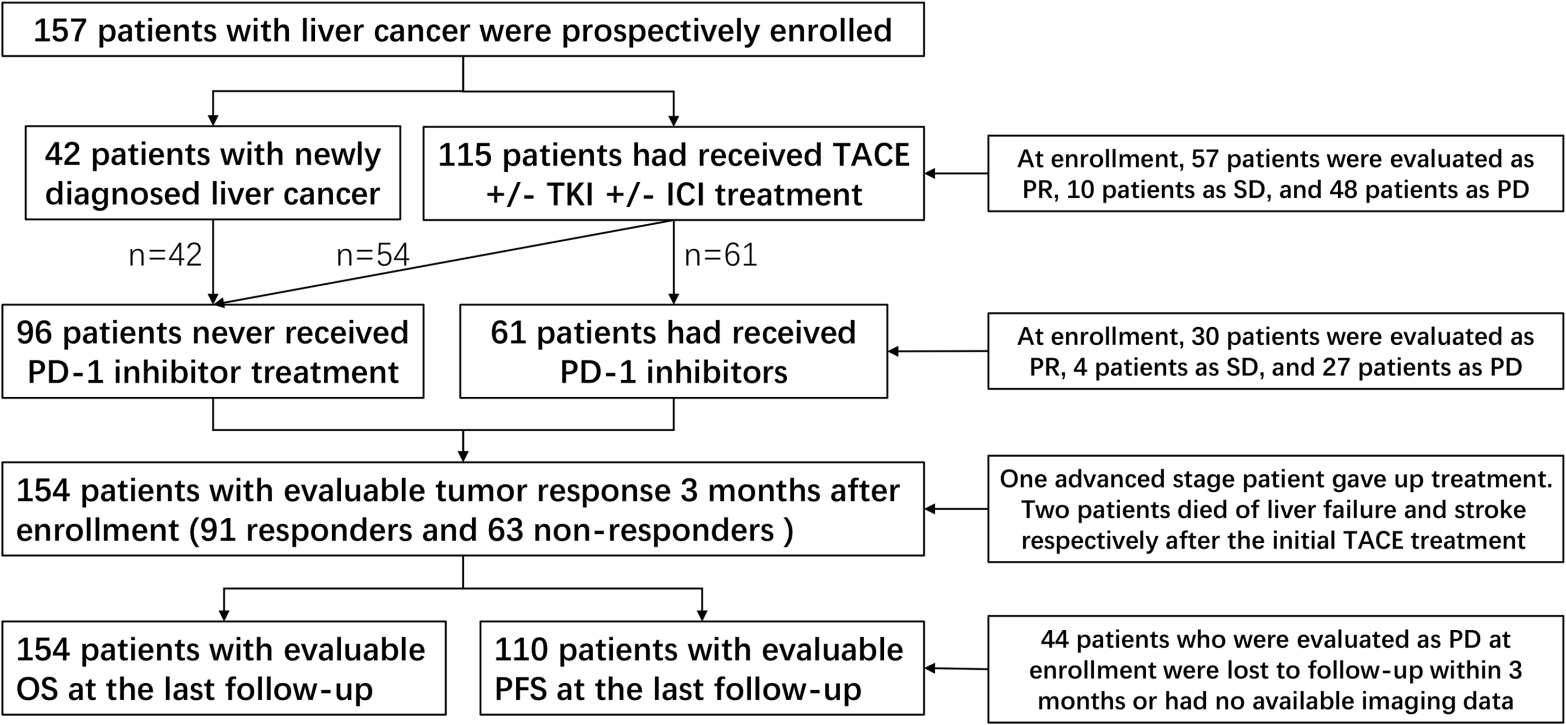
Patient distribution of clinical baseline characteristics and prognosis. TACE, transcatheter arterial chemoembolization; TKI, tyrosine kinase inhibitors; ICI, immune checkpoint inhibitor; PD-1, programmed cell death-1; PR, partial response; SD, stable disease; PD, progressive disease.

From September 2021, a total of 157 patients were enrolled within 6 months and followed up until June 2023. These included 115 previously treated patients and 42 newly diagnosed patients. At enrollment, compared with those patients who had not undergone PD-1 inhibitor treatment (n = 96), those who had received this treatment (n=61) had advanced stage disease, multiple nodules, and extrahepatic metastasis characteristics (all p < 0.05) (**Table 1**). One patient with advanced-stage HCC opted to discontinue treatment, while the remaining patients received TACE-based treatment after enrollment. Among them, 84 patients received TKI+PD-1 inhibitor combination therapy, 30 patients received TKI combination therapy, 11 patients received PD-1 inhibitor combination therapy. Two patients died of liver failure and stroke following an initial TACE treatment. Therefore, the three patients had no assessable or acceptable tumor response or PFS. Additionally, 44 patients who were evaluated as PD at enrollment were lost to follow-up within 3 months or had no available imaging data. Accordingly, we were able to obtain OS data for 154 patients with an evaluable tumor response and evaluable PFS data for 110 patients. The patient distribution of clinical baseline characteristics and prognosis is shown in **Figure 1**.

**Table 1:** Clinical characteristics of patients who had or had not undergone PD-1 inhibitor treatment.

		PD-1 inhibitor treatment		p
		No	Yes	
Age	<56	42	34	0.143
	≥56	54	27	
Sex	Man	87	59	0.145
	Woman	9	2	
Current tumor response ^a^	PR	27	30	0.93
	SD+PD	27	31	
Child-Pugh class	A	65	38	0.646
	B	25	20	
	C	6	3	
BCLC stage	A	22	5	0.01
	B	27	12	
	C	47	44	
Tumor boundary	Clear	50	31	0.877
	Obscure	46	30	
Tumor number	1	19	6	0.003
	2-3	41	15	
	≥4	36	40	
Tumor size (cm)	1-5	41	26	0.795
	5-10	29	21	
	≥10	26	14	
Vascular invasion	No	56	29	0.186
	Yes	40	32	
Extrahepatic metastasis	No	72	35	0.021
	Yes	24	26	

a One hundred and fifteen patients who had previously received treatment had evaluable tumor responses at enrollment.

PR, partial response; SD, stable disease; PD, progressive disease.

### 3.2 Clinical intergroup differences in PD-1+ T cells

**Figure 2:**
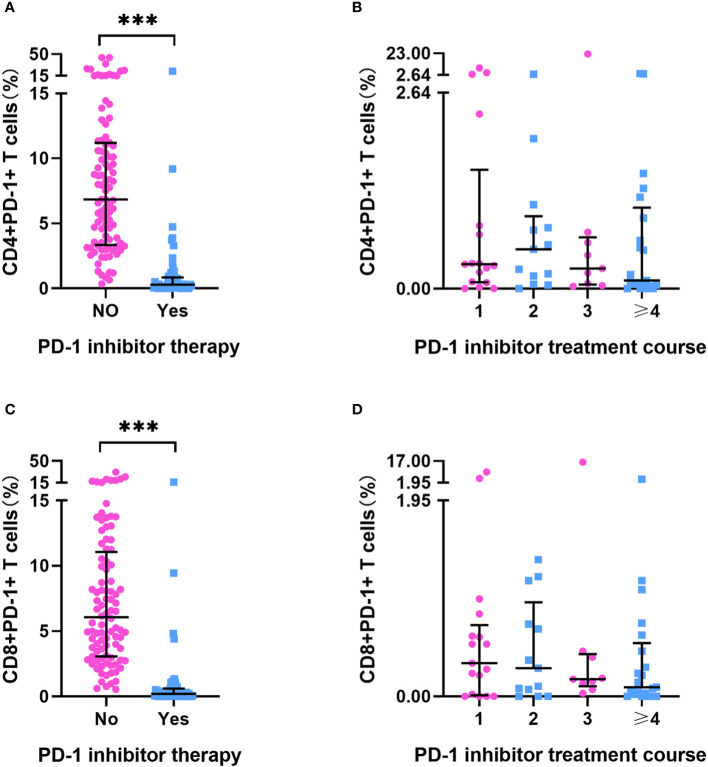
PD-1+ T cell frequency in HCC patients. **(A)** The frequency of CD4+PD-1+T cells in HCC patients treated with or without PD-1 inhibitor (No=96, Yes=60). **(B)** The frequency of CD4+PD-1+T cells in HCC patients after different PD-1 inhibitor treatment courses (once [n=17], twice [n=13], three times [n=9], ≥ four times [n=21]). **(C)** The frequency of CD8+PD-1+T cells in HCC patients treated with or without PD-1inhibitor treatment (No=96, Yes=60). **(D)** The frequency of CD8+PD-1+T cells in HCC patients after different PD-1 inhibitor treatment courses (once [n=17], twice [n=13], three times [n=9], ≥ four times [n=21]). ***p < 0.001.

**Figure 3:**
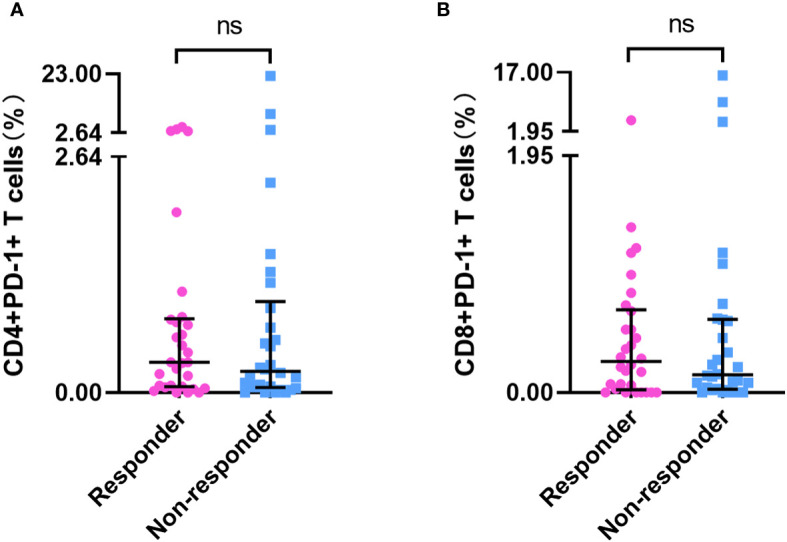
PD-1+ T cell frequency in responders and non-responders who had undergone PD-1 inhibitor treatment. The frequency of CD4+PD-1+ T cells **(A)** and CD8+PD-1+ T cells **(B)** in PD-1 inhibitor responders (n=30) and non-responders (n=30). ‘ns’ means No significance.

Baseline PD-1+ T-cell frequency data was obtained for 156 patients. Compared with those in patients who had not received PD-1 inhibitor treatment (n=96), we detected approximately 20- to 30-fold reductions in the frequencies of CD4+PD-1+ T cells and CD8+PD-1+ T cells in patients who had received PD-1 inhibitor treatment (n = 60) (p < 0.001) (**Supplementary Table 1**, **Figures 2A, C**). However, we detected no differences among the subgroups with different ICI treatment courses (p > 0.05) (**Figures 2B, D**), which means that the PD-1 receptor on the surface of T cells can be effectively blocked after a single administration of PD-1 inhibitor. In patients who had been treated with PD-1 inhibitors, there were no significant differences in the percentages of CD4+PD-1+ T cells and CD8+PD-1+ T cells between the responder (n=30) and non-responder (n=30) groups at enrollment (p > 0.05) (**Figure 3**). This suggests that PD-1+ T cell levels did not rebound in patients who did not respond to PD-1 inhibitors.

In all 156 patients, we detected extremely high percentages of CD4+PD-1+ T cells and CD8+PD-1+ T cells in several patients (> median plus triple interquartile range; **Figures 2**, **3**). We have known that PD-1 inhibitors can significantly reduce the level of PD-1+ T cells. Therefore, we first analyzed the reasons for the abnormal value of PD-1+ T cells in patients who have been treated with PD-1 inhibitors. In these patients, seven (4 responders and 3 non-responders) had an extremely high CD4+PD-1+ T-cell frequency (> 2.64%). Among the four responders, one patient had a 5-month interval from the final administration of sintilimab and the other three patients had a lower CD4+PD-1+ T-cell frequency than did those who had not undergone PD-1 inhibitor treatment. Among the three non-responders, two had a 2-/3-month interval from the final dose of camrelizumab. We also identified four patients with an extremely high CD8+PD-1+ T-cell frequency (> 1.95%), one of whom was a responder (with a 5-month interval from the final use of sintilimab), whereas the remaining three were non-responders (with one patient having a 3-month interval from the final use of camrelizumab). According to our case analysis, prolonged PD-1 inhibitor treatment intervals led to an increased rebound in the frequency of PD-1+ T cells. IL-6 and IFN-γ were reported to regulate the expression of PD-1 and PD-L1 in the tumor microenvironment. Therefore, we further analyzed their correlation with PD-1+ T cells in patients who did not receive PD-1 inhibitor treatment. In these patients, no significant correlations were detected between CD4+PD-1+ T cells and CD8+PD-1+ T cells and IL-6 or IFN-γ (p < 0.05) (**Supplementary Table 2**).

### 3.3 Prognostic analysis of tumor response in patients with different lymphocyte subsets and cytokines levels

When assessed at 3 months after enrollment, 154 patients (91 responders and 63 non-responders) had evaluable tumor responses. The tumor response (responder vs. non-responder) significantly differentiated the survival benefits with respect to PFS (hazard ratio [HR] = 2.968, 95% confidence interval [CI] = 1.710-5.150, p < 0.001) and OS (HR = 5.110, 95% CI = 3.111-8.392, p < 0.001) (**Supplementary Figure 2**). Subsequent ROC curve analysis of 20 lymphocyte subsets and the cytokines IL-6 and IFN-γ based on tumor response revealed significant differences in CD3+ T-cell counts, CD4+ T-cell counts, CD8+ T-cell counts, CD8+CD28+ T-cell frequency, CD8+CD38+ T-cell frequency, CD8+PD-1+/CD4+PD-1+ T-cell ratio, and the concentration of IL-6 in predicting tumor response (all p < 0.05) (**Supplementary Table 3**). The patients were divided into high and low groups based on the Youden indices of the aforementioned lymphocyte subsets and the cytokine IL-6, as well as the median of other previously widely assessed immune indicators, including NK cell counts, NK cell frequency, CD8+/CD4+ T-cell ratio, CD8+CD28− T-cell frequency, CD4+PD-1+ T-cell frequency, CD8+PD-1+ T-cell frequency, and IFN-γ.

**Figure 4:**
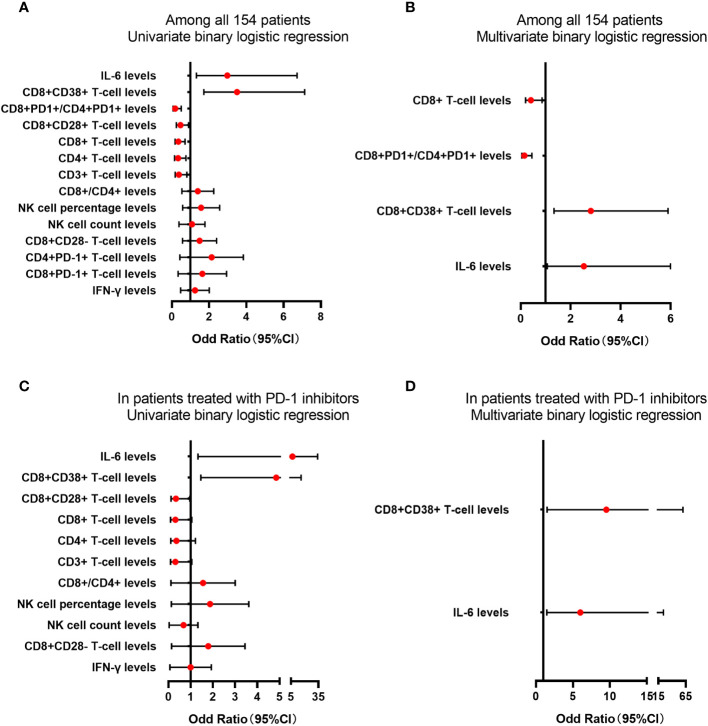
The analysis of lymphocyte subsets and cytokine levels in predicting the tumor response. The lymphocyte subsets and cytokines levels in predicting the tumor response for PD-1 treatment in univariate **(A)** or multivariate **(B)** binary logistic regression among all 154 patients. The lymphocyte subsets and cytokines levels in predicting the tumor response for PD-1 treatment in univariate **(C)** or multivariate **(D)** binary logistic regression in patients treated with PD-1 inhibitors.

The predictive factors in the ROC curve analysis similarly revealed significant differences in the univariate logistic regression analysis (all p<0.05). Multivariate logistic regression analysis indicated significant differences in CD8+ T-cell counts (odds ratio [OR] = 0.409, 95% CI = 0.196-0.855, p = 0.018), CD8+CD38+ T-cell frequency (OR = 2.806, 95% CI = 1.335-5.898, p = 0.006), CD8+PD-1+/CD4+PD-1+ T-cell ratio (OR = 0.149, 95%CI = 0.05-0.451, p = 0.001), and IL-6 (OR = 2.527, 95%CI = 1.065-5.992, p = 0.035) (**Figures 4A, B**). Furthermore, univariate logistic regression analysis performed for the subgroup of patients who had undergone PD-1 inhibitor treatment, revealed that there were significant differences in CD8+CD28+ T-cell frequency, CD8+CD38+ T-cell frequency, and IL-6 (all p < 0.05), whereas multivariate analysis revealed significant differences in CD8+CD38+ T-cell frequency (OR = 5.997, 95%CI = 1.470-24.471, p = 0.013) and IL-6 (OR = 9.525, 95%CI = 1.509-60.127, p = 0.011) (**Figures 4C, D**). Moreover, we obtained evaluable tumor responses for 34 patients who commenced PD-1 inhibitor treatment following enrolment. On the basis of univariate analysis, we detected no significantly difference between responder and non-responder groups with respect to the baseline frequencies of CD4+PD-1+ T cells, CD8+PD-1+ T cells, or the ratio of CD8+PD-1+/CD4+PD-1+ T cells (all p > 0.05).

### 3.4 Survival analysis of RFS and OS in patients with different lymphocyte subsets and cytokines levels

Among the 110 patients with evaluable PFS, univariate survival analysis revealed a significant difference in the levels of six T-cell subsets and IL-6 (all p < 0.05), and multivariate analysis revealed significant differences in the levels of CD4+ T cells (≤ 268/μL vs. >268/μL) (HR = 0.357, 95%CI = 0.190-0.672, p = 0.01), NK cells (≤ 167/μL vs. >167/μL) (HR = 0.374, 95%CI = 0.209-0.670, p = 0.001), and CD8+CD28+ T cells (≤ 64% vs. >64%) (HR = 0.579, 95%CI = 0.345-0.971, p = 0.038) (**Table 2**). Among 35 patients who had undergone PD-1 inhibitors treatment, univariate analysis revealed significant differences in the levels of CD3+ T cells, CD4+ T cells, NK cells, and CD8+CD28+ T cells (all p < 0.05), whereas the multivariate analysis revealed differences in the levels of CD4+ T cells (≤ 268/μL vs. >268/μL) (HR = 0.242, 95%CI = 0.077-0.762, p = 0.015), NK cells (≤ 167/μL vs. >167/μL) (HR = 0.332, 95%CI = 0.106-1.042, p = 0.059), and CD8+CD28+ T cells (≤ 64% vs. >64%) (HR = 0.331, 95%CI = 0.113-0.971, p = 0.044) (**Supplementary Table 4**). Furthermore, among patients who commenced PD-1 inhibitor treatment following enrollment, 28 had evaluable PFS, and with the exception of CD4+PD-1+ T cell levels (HR: 2.401, 95%CI=1.000-5.770, p=0.05), univariate analysis revealed no significant differences among these patients with respect to the levels of CD8+PD-1+ T cells (HR = 1.326, p = 0.504) and CD8+PD-1+/CD4+PD-1+ T-cell ratio (HR = 0.658, p = 0.364).

**Table 2:** Survival analysis for progression-free survival.

All patients	Groups	N	Univariate survival analysis		Multifactor survival analysis	
			HR (95% CI)	p	HR (95% CI)	p
CD3+ T cells (/μL)	≤ 533	21	1			
	>533	88	0.31 (0.176-0.545)	<0.001		
CD4+ T cells (/μL)	≤ 268	17	1		1	
	>268	92	0.214 (0.118-0.388)	<0.001	0.357 (0.190-0.672)	0.001
CD8+ T cells (/μL)	≤219	30	1			
	>219	79	0.390 (0.232-0.655)	<0.001		
CD8+/CD4+ ratio	≤ 0.63	58	1			
	>0.63	51	1.178 (0.713-1.947)	0.522		
NK cells (%)	≤ 17	56	1			
	>17	49	0.511 (0.299-0.872)	0.014		
NK cells (/μL)	≤ 167	49	1		1	
	>167	56	0.303 (0.176-0.520)	<0.001	0.374 (0.209-0.670)	0.001
CD8+CD28+ T cells (%)	≤ 64	48	1		1	
	>64	62	0.587 (0.356-0.967)	0.036	0.579 (0.345-0.971)	0.038
CD8+CD28− T cells (%)	≤ 9	59	1			
	>9	51	1.130 (0.686-1.863)	0.631		
CD8+CD38+ T cells (%)	≤ 59	54	1			
	>59	56	1.507 (0.906-2.504)	0.114		
CD4+PD-1+ T cells (%)	≤ 7	41	1			
	>7	34	1.511 (0.835-2.733)	0.172		
CD8+PD-1+ T cells (%)	≤ 6	38	1			
	>6	37	0.922 (0.510-1.667)	0.788		
CD8+PD-1+/CD4+PD-1+ ratio	≤ 0.55	13	1			
	>0.55	62	0.560 (0.276-1.135)	0.107		
IL-6 (pg/mL)	≤ 25	91	1			
	>25	19	2.351 (1.324-4.173)	0.004		
IFN-γ (pg/mL)	≤ 2	61	1			
	>2	49	0.915 (0.554-1.512)	0.728		

After ROC analysis, patients were divided into high and low groups based on the Youden index or median of lymphocyte subsets and cytokines (**Supplementary Table 3**). PD-1+ T cells were only analyzed in patients who had not undergone PD-1 inhibitor treatment.

Among the 154 patients with evaluable OS, univariate survival analysis revealed significant differences in the levels of 10 peripheral blood immune indicators, including CD8+/CD4+ T-cell ratio, CD8+PD-1+/CD4+PD-1+ T-cell ratio, and IL-6 (all p < 0.05), whereas multivariate analysis revealed significant differences in the levels of CD4+ T cells (≤ 268/μL vs. >268/μL) (HR = 0.433, 95%CI = 0.198-0.948, p = 0.036), CD8+ T cells (≤ 219/μL vs. >219/μL) (HR = 0.505, 95%CI = 0.312-0.814, p = 0.005), NK cells (≤ 167/μL vs. >167/μL) (HR = 0.569, 95%CI = 0.345-0.939, p = 0.027), CD8+CD28+ T cells (≤ 64% vs. >64%) (HR = 0.403, 95%CI = 0.241-0.675, p = 0.001), and IL-6 (≤ 25pg/mL vs. >25pg/mL) (HR = 2.036, 95%CI = 1.208-3.432, p = 0.008) (**Table 3**). Among 61 patients who had undergone PD-1 inhibitor treatment, univariate survival analysis of OS revealed significant differences in the levels of CD8+CD28+ T cells (p < 0.05), IL-6 (p < 0.05), and NK cells (p = 0.057), and, consistently, multivariate analysis revealed similar significant differences in the three parameters (HR = 0.464, 95%CI = 0.216-1.000, p = 0.05; HR = 3.307, 95%CI = 1.500-7.287, p = 0.003; HR = 0.455, 95%CI = 0.207-0.997, p = 0.049) (**Supplementary Table 5**). Among patients who commenced PD-1 inhibitor treatment following enrollment, 34 had an evaluable OS, and for these individuals, univariate analysis revealed no significant differences in the levels of CD4+PD-1+ T cells, CD8+PD-1+ T cells, or CD8+PD-1+/CD4+PD-1+ T-cell ratio (all p > 0.05).

**Table 3:** Survival analysis for overall survival.

All patients	Groups	N	Univariate survival analysis		Multifactor survival analysis	
			HR (95% CI)	p	HR (95% CI)	p
CD3+ T cells (/μL)	≤ 533	38	1			
	>533	114	0.421 (0.261-0.680)	<0.001		
CD4+ T cells (/μL)	≤ 268	31	1		1	
	>268	121	0.361 (0.220-0.591)	<0.001	0.433 (0.198-0.948)	0.036
CD8+ T cells (/μL)	≤219	51	1		1	
	>219	101	0.453 (0.285-0.718)	0.001	0.504 (0.312-0.814)	0.005
CD8+/CD4+ ratio	≤ 0.63	76	1			
	>0.63	77	1.631 (1.024-2.596)	0.039		
NK cells (%)	≤ 17	76	1			
	>17	70	0.776 (0.483-1.247)	0.295		
NK cells (/μL)	≤ 167	73	1		1	
	>167	73	0.489 (0.301-0.796)	0.004	0.569 (0.345-0.939)	0.027
CD8+CD28+ T cells (%)	≤ 64	79	1		1	
	>64	74	0.366 (0.223-0.600)	<0.001	0.403 (0.241-0.675)	0.001
CD8+CD28− T cells (%)	≤ 9	75	1			
	>9	78	1.661 (1.041-2.651)	0.033		
CD8+CD38+ T cells (%)	≤ 59	63	1			
	>59	90	1.782 (1.080-2.938)	0.024		
CD4+PD-1+ T cells (%)	≤ 7	47	1			
	>7	46	1.783 (0.933-3.408)	0.08		
CD8+PD-1+ T cells (%)	≤ 6	46	1			
	>6	47	0.884 (0.471-1.657)	0.7		
CD8+PD-1+/CD4+PD-1+ ratio	≤ 0.55	22	1			
	>0.55	71	0.376 (0.197-0.719)	0.003		
IL-6 (pg/mL)	≤ 25	121	1		1	
	>25	31	2.600 (1.592-4.245)	<0.001	2.036 (1.208-3.432)	0.008
IFN-γ (pg/mL)	≤ 2	84	1			
	>2	68	1.017 (0.641-1.615)	0.942		

After ROC analysis, patients were divided into high and low groups based on the Youden index or median of lymphocyte subsets and cytokines (**Supplementary Table 3**). PD-1+ T cells were only analyzed in patients who had not undergone PD-1 inhibitor treatment.

### 3.5 Correlation between independent prognostic factors and clinical characteristics

**Figure 5:**
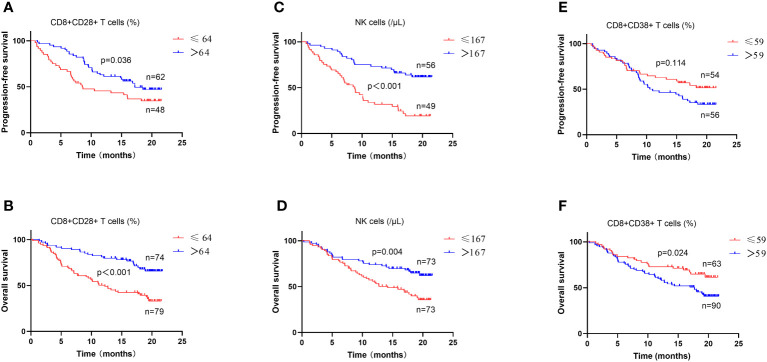
Survival analysis in patients with different lymphocyte subsets and cytokines levels. The levels of CD8+CD28+ T cells **(A, B)**, NK cells **(C, D)** and CD8+CD38+T cells **(E, F)** were analyzed for progression-free survival and overall survival in all patients.

Among all patients and the subgroup of patients treated with PD-1 inhibitors, CD8+CD28+ T cells and NK cells were identified as the common independent prognostic factors for PFS and OS (**Figure 5**), whereas CD8+CD38+ T cells were common independent prognostic factors for tumor response. Correlation analysis performed to assess the significance of these three factors from a clinical perspective indicated associations with characteristic features of tumor progression, including advanced tumor stage, blurred boundaries, larger tumors, multiple nodules, extrahepatic metastasis, vascular invasion, and poor liver function (all p < 0.05) (**Table 4**).

**Table 4:** Correlations between independent prognostic factors and clinical characteristics.

		CD8+CD28+ T cell (%)			NK cell (cells/μL)			CD8+CD38+ T cell (%)		
		low	high	p	low	high	p	low	high	p
Age	<56	33	43	0.079	41	32	0.163	26	50	0.126
	≥ 56	46	34		34	42		37	43	
Sex	Man	76	69	0.108	68	71	0.198	61	84	0.12
	Woman	3	8		7	3		2	9	
Current tumor response ^a^	PR	25	32	0.061	28	26	0.636	27	30	0.054
	SD+PD	35	22		31	24		17	40	
Child-Pugh class	A	45	58	0.036	40	58	0.005	48	55	0.083
	B	27	17		30	13		12	32	
	C	7	2		5	3		3	6	
BCLC stage	A	8	19	0.013	9	18	0.056	21	6	<0.001
	B	17	22		17	21		18	21	
	C	54	36		49	35		24	66	
Tumor boundary	Clear	34	46	0.037	31	46	0.011	38	42	0.063
	Obscure	45	31		44	28		25	51	
Tumor number	1	7	18	0.006	10	15	0.316	19	6	<0.001
	2-3	25	31		25	28		23	33	
	≥4	47	28		40	31		21	54	
Tumor size (cm)	1-5	29	37	0.046	26	38	0.079	38	28	0.001
	5-10	23	27		25	22		14	36	
	≥10	27	13		24	14		11	29	
Vascular invasion	No	34	50	0.006	35	48	0.025	45	39	<0.001
	Yes	45	27		40	26		18	54	
Extrahepatic metastasis	No	48	59	0.033	46	56	0.06	54	53	<0.001
	Yes	31	18		29	18		9	40	

a Patients who had previously received treatment had evaluable tumor responses at enrollment.

PR, partial response; SD, stable disease; PD, progressive disease.

### 3.6 Associations between changes in independent prognostic factors and tumor response

**Figure 6:**
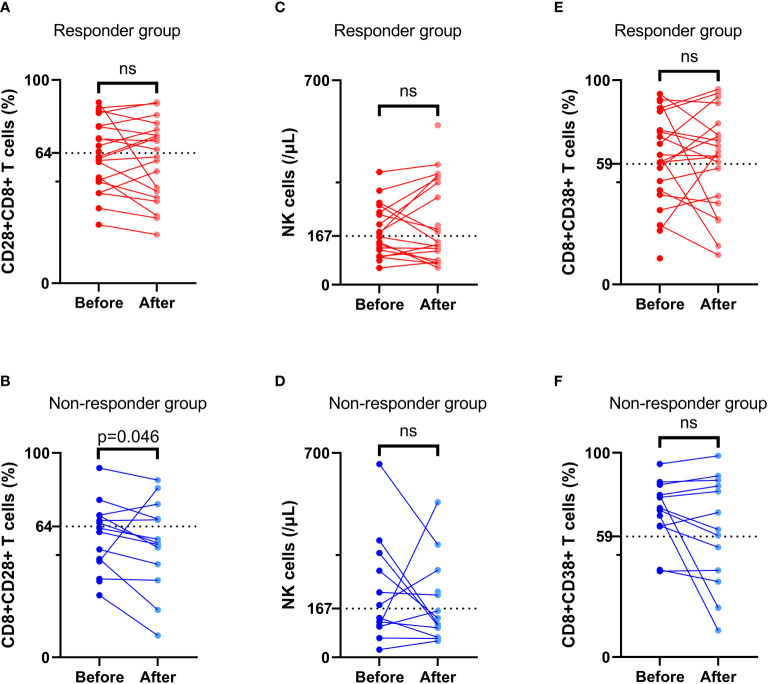
Comparison of lymphocyte subsets before and after treatment. The frequency of CD8+CD28+ T cells **(A, B)**, NK cell number **(C, D)**, and the frequency of CD8+CD38+ T cells **(E, F)** before and after treatment in the response group and non-response group. ‘ns’ means No significance.

**Figure 7:**
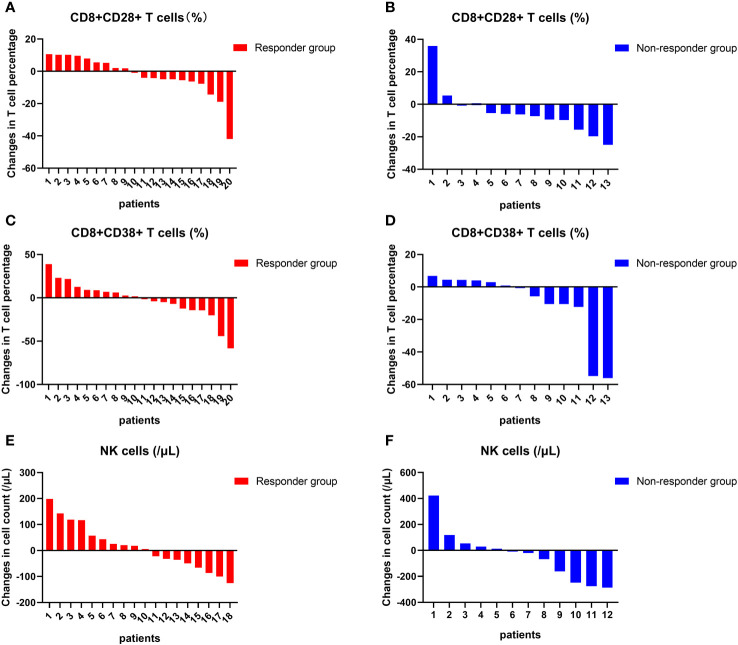
Changes in lymphocyte subsets in individuals. The changes in the frequency of CD8+CD28+ T cells **(A, B)**, NK cell counts **(C, D)**, and the frequency of CD8+CD38+ T cells **(E, F)** at the individual level before and after treatment in the response group and non-response group.

At 4 to 8 weeks post-enrollment, we re-assessed lymphocyte subsets in 33 patients and analyzed associations between the changes in the aforementioned three independent prognostic factors and tumor response. With respect to the non-responder subgroup (n=13), we accordingly detected a significant reduction in CD8+CD28+ T-cell frequency after 4–8 weeks (p = 0.046). Contrastingly, in neither subgroup did we detect any significant differences regarding comparisons of other indicators measured before and after treatment (**Figure 6**). In addition, there were no significant differences between the responder and non-responder subgroups with respect to changes in the three independent prognostic factors (all p > 0.05) (**Figure 7**), thereby tending to indicate that changes in these factors would not be effective as predictors of a tumor response.

## 4 Discussion

To date, few studies have sought to verify the prognostic efficacy of peripheral blood lymphocyte subsets after TACE for liver cancer. However, given the significant heterogeneous responses of patients with advanced liver cancer treated with a combination of ICIs and an overall poor prognosis, accurate predictive markers are urgently needed. In this prospective study, we identified the levels of CD8+CD28+ T cells and NK cells as being independent prognostic factors for PFS and OS in all patients, as well as in subgroups of patients treated with PD-1 inhibitors. Furthermore, CD8+CD38+ T cells were found to be independent prognostic factors for tumor response. However, although PD-1 inhibitors can significantly block PD-1 receptors on the surface of T cells, we established that neither the baseline levels of CD8+PD-1+ and CD4+PD-1+ T cells nor the ratio or changes in these cells would serve as effective predictors of the prognosis of patients with liver cancer.

PD-1 receptors expressed on the surface of hematopoietic cells have an inhibitory function involving the negative regulation of immune responses, particularly in response to tumors. PD-1/PD-L1 blocking antibodies have been demonstrated to reverse these inhibitory effects and have accordingly shown clinical benefits in the treatment of tumors. However, in clinical practice, patients treated with PD-1 inhibitors often exhibit advanced tumor characteristics, making it difficult to predict their clinical benefit. Given that the expression of PD-1 on TILs has been established to be associated with prognosis in HCC, we sought to assess the utility of peripheral lymphocyte cells, including PD-1+ T cells, as biomarkers for predicting disease progression and prognosis in patients with HCC. We found that the baseline frequencies of circulating CD4+PD-1+ and CD8+PD-1+ T cells in HCC patients were 6.85% and 6.08%, respectively, whereas in patients treated with PD-1 inhibitors, we detected significantly reduced values of 0.30% and 0.21%, respectively, thereby indicating that PD-1 inhibitors can effectively block PD-1 receptors on the surface of T cells. The expression of PD-1 on circulating T cells in healthy individuals is lower than that in patients with NSCLC (30), and an increase in PD-1 expression is associated with tumor staging (31, 32). In the present study, however, we detected no significant correlation between PD-1 expression and tumor stage or liver function, except for patients with high levels of CD8+PD-1+ T cells associated with multiple tumors (data not shown). A high frequency of CD8+PD-1+TILs has previously been found to be a predictor of tumor responses in patients receiving ICI treatment. In contrast, a high frequency of circulating CD8+PD-1+ T cells is considered to be indicative of tumor progression in patients receiving sorafenib treatment (33). This indicates the contrasting predictive performance of PD-1+ T cells in relation to the therapeutic efficacy of ICI and TKI. The findings of a further study have revealed an increase in the expression of PD-1 in patients with recurrent NSCLC treated with PD-1 inhibitors (30). In the present study, however, we found that with the exception of the levels of CD4+PD-1+ T cells, which were shown to predict a worse OS in univariate analysis of the subgroup of patients treated with PD-1 inhibitors, there was no significant association between the baseline levels of PD-1+ T cells and patient prognosis. In patients treated with PD-1 inhibitors, we failed to detect any significant increase in the expression of PD-1 with tumor progression, and, consequently, we speculate that although PD-1 inhibitors can effectively block PD-1 receptors on the surface of T cells, they may not necessarily effectively activate T cell function in these non-responder patients. From another point of view, if the frequency of circulating PD-1+T cells in patients who fail to respond to PD-1 inhibitor treatment is significantly reduced (meaning the blood concentration of PD-1 inhibitor is high enough), we speculate that continuing medication may not be a wise choice. The abnormal increase in PD-1+ T-cell frequencies observed in patients treated with PD-1 inhibitors can partly be attributed to the extended treatment interval. Additionally, IL-6 and IFN-γ are considered to be the prominent stimulators that contribute to the expression of PD-1 and PD-L1 in the tumor microenvironment (34, 35); however, we were unable to detect any significant correlations between IL-6 or IFN-γ and PD-1+ T cells, which would thus tend to indicate that inflammatory cytokines are not associated with the abnormally high percentages of PD-1+ T cells.

Contrary to our expectations, we failed to detect any positive correlation between PD-1+ T cells and patient prognosis, which we assume could be attributable to one or more of the following factors. Firstly, there are differences regarding the expression of PD-1 on lymphocytes in peripheral blood and tumor tissues, and the expression of this protein does not fully reflect the immune status of the body. The frequency of PD-1+ TILs is significantly greater than that of peripheral PD-1+ T cells (33, 36), whereas in contrast, there is no significant difference in the frequency of circulating PD-1high T cells between healthy individuals and cancer patients (33). Secondly, PD-1+ T cells contain a group of cell subsets of differing functional status, and their co-expression with other molecules may represent a group of specific functional cell subsets, which may be more meaningful for identifying patient heterogeneity or predicting prognosis. For example, circulating PD-1+ early effector memory CD8+ T cells (CD28+CD27-CD45RO+) are characterized by early responses to anti-PD-1 therapy in patients with NSCLC (37). Moreover, circulating PD-1+TIGIT+CD8+ T cells are significantly upregulated in patients with HCC and are correlated with an advanced disease stage and poor prognosis (38). Thirdly, PD-1+ T cells are tumor-specific, and PD-1 is more highly expressed on tumor-associated antigen-specific CD8+ TILs than on other CD8+ TILs (36). In some cases, tumor-reactive peripheral blood lymphocytes are characterized by an overexpression of PD-1 receptors (39, 40). However, higher levels of PD-1+ T cells have also been established to be associated with other non-tumor factors, including aging, chronic inflammation, and infection. Consequently, the clinical significance of PD-1 expression on the peripheral lymphocytes of HCC patients needs further evaluation.

Theoretically, PD-1 inhibitors do not directly influence T cell surface receptors such as CD28, CD38, CD16, and CD56. In this study, we identified CD8+CD28+ T and NK cells as independent prognostic factors for PFS and OS in patients treated with TACE with or without the administration of PD-1 inhibitors. Similarly, CD38+ T cells were established to be an independent prognostic factor for tumor response. CD28 receptors are important co-stimulatory signals on the surface of T cells that in response to activation, exert anti-tumor effects when combined with B7 molecules on antigen-presenting cells (41). On the basis of the expression of CD28+, CD8+ T cells were divided into CD8+CD28+ cytotoxic lymphocytes and CD8+CD28− senescent T cells. In this regard, it has previously been demonstrated that circulating levels of CD8+CD28+ T cells are lower in patients with ovarian cancer than in their benign counterparts (42), whereas the findings of a further study have indicated that high levels of circulating CD8+CD28+ T cells can serve as a predictor of immunotherapeutic responses and a more favorable prognosis in cancer patients (43). In the present study, we confirmed that high levels of circulating CD8+CD28+ T cells are associated with tumor response and prolonged PFS and OS. In contrast, the loss of CD28 is associated with a reduced proliferation of CD8+ T cells and a less efficient ability to recognize diverse antigens. It has also previously been found that in patients with lung cancer, circulating CD28−CD57+KLRG1+CD8+ T cells were associated with a lack of benefit from ICIs (44). Our findings similarly indicate that elevated levels of CD8+CD28− T-cell expression are associated with a poor OS in all patients, although are not predictive of tumor response or PFS, neither did they have an effective prognostic value in the PD-1 inhibitor treatment subgroup. In summary, CD8+CD28+ T cells are identified as an important functional subgroup that warrants further research.

CD38 was initially considered a biomarker for identifying activated T cells and thymocytes (45). In recent years, however, it has been established that CD38 is a member of the ribosyl cyclase family of proteins that is widely expressed on the surface of non-hematopoietic cells and several types of immune cells, in which it plays roles in adenosine synthesis, thereby contributing to immune escape (46). In HCC, a high frequency of CD38+PD-1+CD8+ T cells is associated with high histopathological grades (III and IV), thereby indicating that CD38, a T-cell co-exhaustion marker, is linked to tumor aggressiveness (27). A recent study has revealed that a heightened level of CD38 expression in TILs promotes increases in the levels of Ki-67 in tumor cells, and that highly expressed CD38+ TILs independently predict shorter OS and PFS (47). The findings of further studies have indicated that the progression of prostate cancer is associated with increases in CD38+ tumor-infiltrating immune cell density, which is independently associated with a worse OS (48). Moreover, anti-CD38 antibodies have been shown to enhance tumor inhibition, and in several clinical trials, have been found to have certain clinical benefits for patients with tumors (49, 50). Our findings in the present study also confirmed that high levels of CD38+CD8+ T cells are indicative of incomplete tumor response in patients with HCC treated with TACE with or without the administration of PD-1 inhibitors.

NK cells are part of the body’s first line of defense against cancer cells and viral infection that can directly and non-specifically kill tumor cells and, as such, these cells have been extensively studied. The association between these lymphocytes and tumor prognosis has been reported in many clinical studies. For example, thermal ablation has been demonstrated to promote increases in the frequency and function of CD3-CD56+NK cells in the peripheral blood of patients with HCC, and is associated with recurrence-free survival (51), whereas radiation therapy has been found to have a significant effect on the levels of peripheral NK and NKT-like cells, with a higher percentage of NKT-like cells being found to be associated with a longer OS in HCC patients (52). Furthermore, sorafenib has been observed to modify the proportion and function of peripheral NK cells, which are associated with treatment outcomes in patients with HCC (53). However, whereas a high frequency of circulating NK cells has been established to be a predictor of tumor response in patients with NSCLC treated with immunotherapy (54), in the present study, although we found NK cells can serve as a predictor of long-term PFS and OS, they showed no significant association with a short-term tumor response. A plausible explanation for this contrasting performance is that circulating NK cells reflect systemic immunity and can predict long-term prognosis, whereas TACE has a significant influence on the tumor response, which may interfere with the short-term predictive performance of NK cells.

From a clinical perspective, we found that the levels of CD8+CD28+ T, NK, and CD8+CD38+ T cells were associated with tumor characteristics and liver function, which, to some extent, would explain their prognostic value. However, we also established that these dynamic changes were relatively ineffective as follow-up indicators for predicting a tumor response. In addition to these independent prognostic factors, IL-6 and CD4+ and CD8+ T cells have been reported as prognostic biomarkers in many studies. For example, the baseline CD4+/CD8+ T-cell ratio and its changes have been identified as prognostic markers for cancer patients (33, 55), although the findings of a recent study have provided evidence to indicate that the CD8+PD-1+ to CD4+PD-1+ T-cell ratio, rather than the CD8+/CD4+ T-cell ratio, is associated with clinical benefits in advanced NSCLC patients treated with ICIs (56). The CD8+PD-1+/CD4+PD-1+ index proposed in this previous study was based on the premise that high CD8+PD-1+ T cell levels are associated with good prognosis in cancer patients, whereas high CD4+PD-1+ T cell levels are associated with poor clinical outcomes. In the present study, however, we were unable to detect any significant associations between PD-1+ T cells and survival, with the exceptions being CD8+PD-1+/CD4+PD-1+ in predicting tumor response and OS, and CD8+/CD4+ in predicting OS in the univariate analysis of all patients. Consequently, the clinical significance of peripheral blood lymphocyte subsets remains to be determined.

Although in this prospective study, we strived to enroll as many patients as possible who had undergone long-term follow-up, the study does have certain limitations. Firstly, we measured only lymphocyte subsets in peripheral blood. In future studies, to identify more reliable predictive biomarkers and elucidate the underlying mechanisms involved, we plan to evaluate both circulating and infiltrating lymphocytes in patients. Secondly, our flow cytometry antibody staining scheme may have produced negative results owing to its simplicity and insufficient accuracy in the detection of lymphocyte functional subsets. Finally, this was primarily an exploratory study based on real-world clinical practice. Many patients had already received treatment before enrollment, as well as different subsequent combination therapies, thereby introducing a certain level of heterogeneity within the study population.

## 5 Conclusion

Our findings in this study revealed that the levels of circulating CD8+CD38+ T cells, CD8+CD28+ T cells, and NK cells are potential prognostic factors for tumor response and long-term survival in patients with HCC treated with TACE, administered with or without PD-1 inhibitors. Although PD-1 inhibitors can effectively block PD-1 receptors on the surface of T cells, the baseline frequency of PD-1+ T cells and changes in the frequency of these cells were established to have limited prognostic value.

## Data availability statement

The original contributions presented in the study are included in the article/**Supplementary Material**. Further inquiries can be directed to the corresponding authors.

## Ethics statement

The studies involving humans were approved by the Ethics Committee of the First Affiliated Hospital of Sun Yat-Sen University. The studies were conducted in accordance with the local legislation and institutional requirements. The participants provided their written informed consent to participate in this study.

## Author contributions

HW: Conceptualization, Data curation, Formal analysis, Funding acquisition, Investigation, Writing – original draft. HH: Conceptualization, Data curation, Investigation, Methodology, Writing – original draft. TL: Formal analysis, Funding acquisition, Methodology, Supervision, Writing – original draft. YC: Formal analysis, Investigation, Methodology, Writing – original draft. JWL: Data curation, Investigation, Writing – original draft. MH: Formal analysis, Methodology, Supervision, Writing – original draft. JP: Formal analysis, Funding acquisition, Supervision, Writing – original draft. EL: Formal analysis, Funding acquisition, Supervision, Writing – original draft. JPL: Conceptualization, Data curation, Formal analysis, Resources, Supervision, Writing – review & editing. WL: Conceptualization, Data curation, Formal analysis, Funding acquisition, Project administration, Resources, Supervision, Writing – review & editing.
