# Supplementary material for: Peripheral blood lymphocyte subsets predict the efficacy of TACE with or without PD-1 inhibitors in patients with hepatocellular carcinoma: a prospective clinical study
Source: Front Immunol. 2024 Feb 9;15:1325330. doi: 10.3389/fimmu.2024.1325330 (PMC10884244; doi:10.3389/fimmu.2024.1325330)
Supplement: Supplementary file 1 [file DataSheet_1.docx]

# Supplementary Figure 1. Flow cytometry gating strategy.


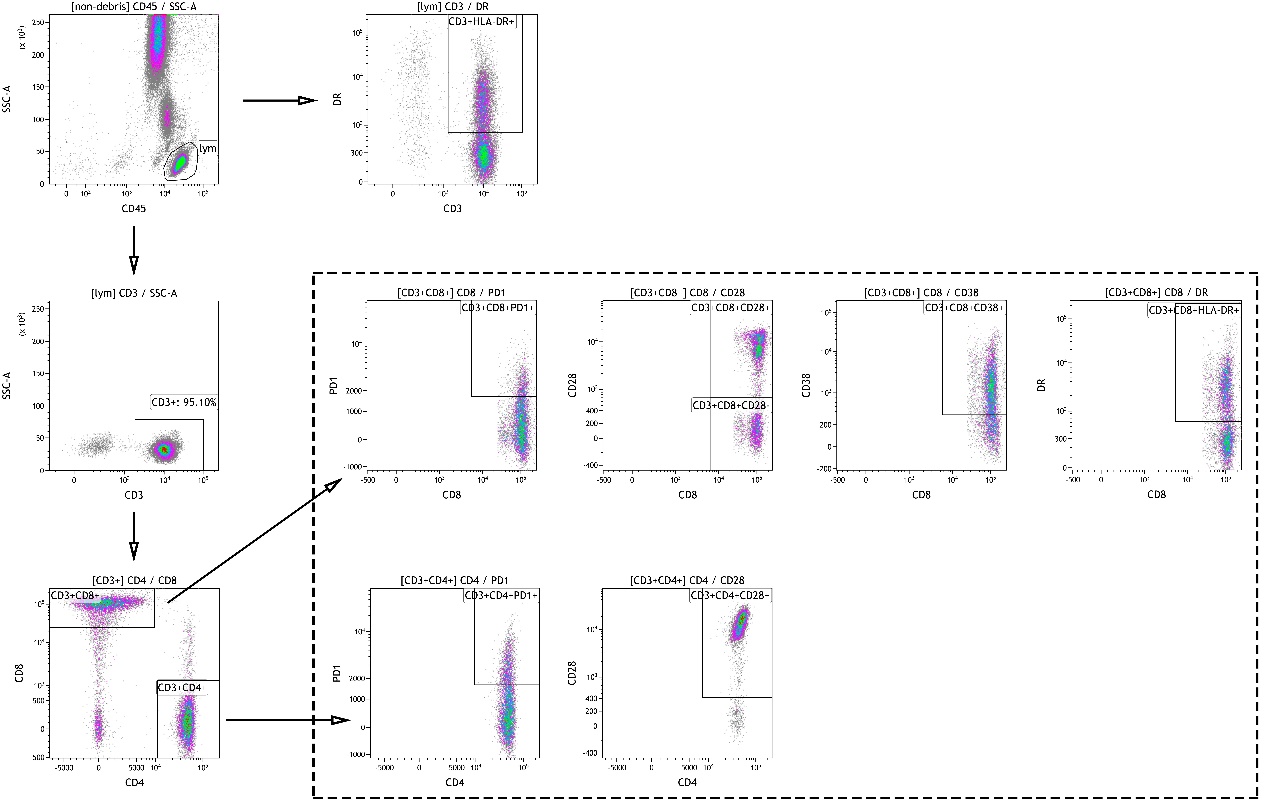


Notes: Firstly, we gated lymphocyte identified by CD45 from leukocyte, then gated CD3+ and CD3+HLA-DR+ T cells from lymphocyte. T cell subsets populations CD4+ and CD8+ was gated from CD3+ T subsets; CD8+PD-1+, CD8+CD28+, CD8+CD28−, CD8+CD38+ and CD8+HLA-DR+ were gated from CD3+CD8+ T subsets (the frequency of CD8+CD28− T cells in this study was determined by calculating the ratio of CD8+CD28− T cells to total lymphocytes [CD8+CD28−/LYM]); CD4+PD-1+ and CD4+CD28+ were gated from CD3+CD4+ T subsets.

# Supplementary Figure 2. Kaplan-Meier curve for progression-free survival (PFS) and overall survival (OS) of responders and non-responders.


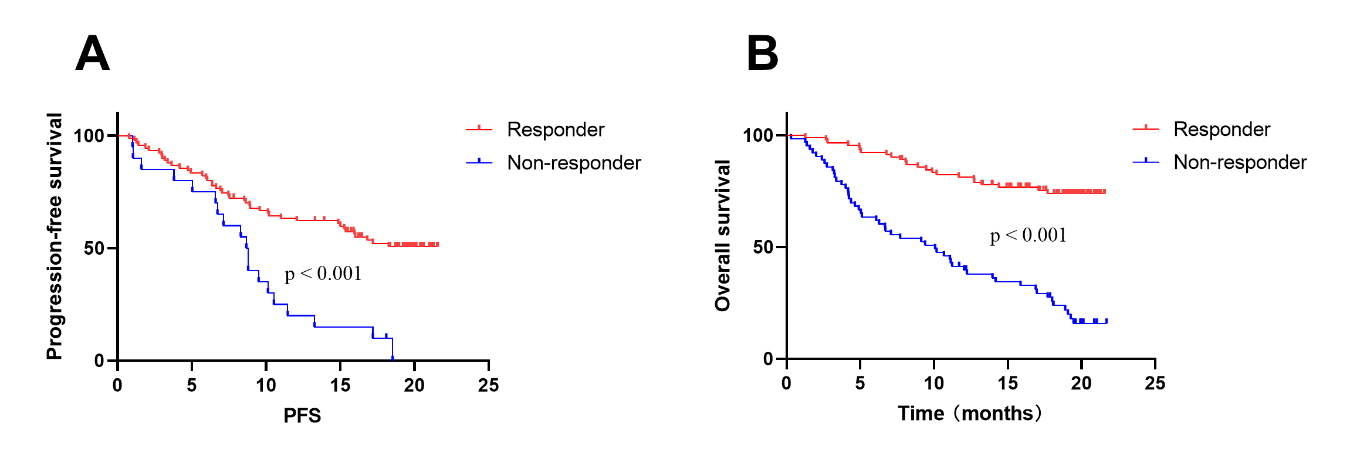


# Supplementary Table 1. Baseline PD-1+ T-cell frequency

|  | PD-1 inhibitors treatment | | P |
| --- | --- | --- | --- |
|  | No (n=96) | Yes (n=60) |  |
| CD4+PD-1+ T cells (%) | 6.85（3.34-11.19） | 0.30（0.06-0.84） | ＜0.001 |
| CD8+PD-1+ T cells (%) | 6.08（3.07-11.06） | 0.21（0.03-0.61） | ＜0.001 |

# Supplementary Table 2. Correlation between PD-1+T cells and cytokines

|  | | IL-6 (pg/mL) | IFN-γ (pg/mL) |
| --- | --- | --- | --- |
| CD4+PD-1+ T cells (%) | Related Coefficient | 0.192 | 0.097 |
|  | p | 0.060 | 0.348 |
| CD4+PD-1+ T cells (%) | Related Coefficient | 0.047 | 0.027 |
|  | p | 0.647 | 0.796 |

Note: Analysis only in patients who had not undergone PD-1 inhibitors.

# Supplementary Table 3. Receiver operating characteristic curve analysis and cut-off value

|  | Predictor | AUC | p | Youden index or Median |
| --- | --- | --- | --- | --- |
| CD3+ T cells (%) | Responder | 0.561 | 0.202 |  |
| CD3+ T cells (/μL) | Responder | 0.606 | 0.026 | 533 |
| CD4+ T cells (%) | Responder | 0.564 | 0.177 |  |
| CD4+ T cells (/μL) | Responder | 0.606 | 0.027 | 268 |
| CD8+ T cells (%) | Responder | 0.511 | 0.811 |  |
| CD8+ T cells (/μL) | Responder | 0.598 | 0.041 | 219 |
| CD8+/CD4+ ratio | Non-Responder | 0.535 | 0.469 | 0.63 |
| NK cells (%) | Non-Responder | 0.55 | 0.307 | 17 |
| NK cells (/μL) | Responder | 0.537 | 0.456 | 167 |
| B cells (%) | Responder | 0.521 | 0.668 |  |
| B cells (/μL) | Responder | 0.564 | 0.192 |  |
| CD4+CD28+ T cells (%) | Responder | 0.581 | 0.088 |  |
| CD8+CD28+ T cells (%) | Responder | 0.601 | 0.034 | 64 |
| CD8+CD28− T cells (%) | Non-Responder | 0.572 | 0.131 | 9 |
| CD3+HLA-DR+ T cells (%) | Non-Responder | 0.554 | 0.259 |  |
| CD8+CD38+ T cells (%) | Non-Responder | 0.659 | ＜0.001 | 59 |
| CD8+HLA-DR+ T cells (%) | Non-Responder | 0.575 | 0.118 |  |
| CD4+PD-1+ T cells (%) | Non-Responder | 0.605 | 0.1 | 7 |
| CD8+PD-1+ T cells (%) | Non-Responder | 0.526 | 0.684 | 6 |
| CD8+PD-1+/CD4+PD-1+ ratio | Responder | 0.64 | 0.028 | 0.55 |
| IL-6 (pg/mL) | Non-Responder | 0.598 | 0.042 | 25 |
| IFN-γ (pg/mL) | Non-Responder | 0.569 | 0.151 | 2 |

Note: PD-1+T cells were only analyzed in patients who had not undergone PD-1 inhibitors.

# Supplementary Table 4. Survival analysis for progression-free survival.

| Patients had undergone PD-1 inhibitors |  |  | Univariate survival analysis | | Multifactor survival analysis | |
| --- | --- | --- | --- | --- | --- | --- |
|  | Groups | N | HR (95% CI) | p | HR (95% CI) | p |
| CD3+ T cells (/μL) | ≤ 533 | 6 | 1 |  |  |  |
|  | ＞533 | 28 | 0.238（0.08-0.708） | 0.01 |  |  |
| CD4+ T cells (/μL) | ≤ 268 | 6 | 1 |  | 1 |  |
|  | ＞268 | 28 | 0.215（0.073-0.634） | 0.005 | 0.242（0.077-0.762） | 0.015 |
| CD8+ T cells (/μL) | ≤219 | 6 | 1 |  |  |  |
|  | ＞219 | 28 | 0.466（0.151-1.442） | 0.185 |  |  |
| CD8+/CD4+ ratio | ≤ 0.63 | 13 | 1 |  |  |  |
|  | ＞0.63 | 21 | 1.039（0.384-2.811） | 0.941 |  |  |
| NK cells (%) | ≤ 17 | 16 | 1 |  |  |  |
|  | ＞17 | 16 | 0.541（0.196-1.489） | 0.234 |  |  |
| NK cells (/μL) | ≤ 167 | 16 | 1 |  | 1 |  |
|  | ＞167 | 16 | 0.239（0.076-0.744） | 0.014 | 0.332（0.106-1.042） | 0.059 |
| CD8+CD28+ T cells (%) | ≤ 64 | 15 | 1 |  | 1 |  |
|  | ＞64 | 20 | 0.32（0.123-0.833） | 0.02 | 0.331（0.113-0.971） | 0.044 |
| CD8+CD28− T cells (%) | ≤ 9 | 14 | 1 |  |  |  |
|  | ＞9 | 21 | 1.398（0.525-3.727） | 0.503 |  |  |
| CD8+CD38+ T cells (%) | ≤ 59 | 15 | 1 |  |  |  |
|  | ＞59 | 20 | 1.001（0.387-2.584） | 0.999 |  |  |
| IL-6 (pg/mL) | ≤ 25 | 30 | 1 |  |  |  |
|  | ＞25 | 5 | 2.305（0.746-7.125） | 0.147 |  |  |
| IFN-γ (pg/mL) | ≤ 2 | 21 | 1 |  |  |  |
|  | ＞2 | 14 | 0.988（0.382-2.553） | 0.98 |  |  |

# Supplementary Table 5. Survival analysis for overall survival.

| Patients had undergone PD-1 inhibitors |  |  | Univariate survival analysis | | Multifactor survival analysis | |
| --- | --- | --- | --- | --- | --- | --- |
|  | Groups | N | HR (95% CI) | p | HR (95% CI) | p |
| CD3+ T cells (/μL) | ≤ 533 | 17 | 1 |  |  |  |
|  | ＞533 | 42 | 0.536（0.268-1.074） | 0.079 |  |  |
| CD4+ T cells (/μL) | ≤ 268 | 16 | 1 |  |  |  |
|  | ＞268 | 43 | 0.518（0.255-1.051） | 0.068 |  |  |
| CD8+ T cells (/μL) | ≤219 | 17 | 1 |  |  |  |
|  | ＞219 | 42 | 0.571（0.286-1.142） | 0.113 |  |  |
| CD8+/CD4+ ratio | ≤ 0.63 | 22 | 1 |  |  |  |
|  | ＞0.63 | 38 | 1.198（0.595-2.413 | 0.612 |  |  |
| NK cells (%) | ≤ 17 | 26 | 1 |  |  |  |
|  | ＞17 | 30 | 0.836（0.422-1.658） | 0.609 |  |  |
| NK cells (/μL) | ≤ 167 | 33 | 1 |  | 1 |  |
|  | ＞167 | 23 | 0.485（0.230-1.023） | 0.057 | 0.455（0.207-0.997） | 0.049 |
| CD8+CD28+ T cells (%) | ≤ 64 | 33 | 1 |  | 1 |  |
|  | ＞64 | 27 | 0.363（0.172-0.765） | 0.008 | 0.464（0.216-1.000） | 0.05 |
| CD8+CD28− T cells (%) | ≤ 9 | 23 | 1 |  |  |  |
|  | ＞9 | 37 | 1.197（0.597-2.400） | 0.612 |  |  |
| CD8+CD38+ T cells (%) | ≤ 59 | 19 | 1 |  |  |  |
|  | ＞59 | 41 | 1.197（0.558-2.566） | 0.644 |  |  |
| IL-6 (pg/mL) | ≤ 25 | 47 | 1 |  | 1 |  |
|  | ＞25 | 12 | 3.192（1.546-6.591） | 0.002 | 3.307（1.500-7.287） | 0.003 |
| IFN-γ (pg/mL) | ≤ 2 | 37 | 1 |  |  |  |
|  | ＞2 | 22 | 1.097（0.549-2.191） | 0.794 |  |  |
